# Biomimetic Magnetoliposomes as Oxaliplatin Nanocarriers: In Vitro Study for Potential Application in Colon Cancer

**DOI:** 10.3390/pharmaceutics12060589

**Published:** 2020-06-24

**Authors:** Beatriz Garcia-Pinel, Ylenia Jabalera, Raul Ortiz, Laura Cabeza, Concepción Jimenez-Lopez, Consolación Melguizo, Jose Prados

**Affiliations:** 1 Institute of Biopathology and Regenerative Medicine (IBIMER), Center of Biomedical Research (CIBM), University of Granada, 18100 Granada, Spain; beatrizgarnel@ugr.es (B.G.-P.); roquesa@ugr.es (R.O.); lautea@ugr.es (L.C.); jcprados@ugr.es (J.P.); 2 Department of Anatomy and Embriology, Faculty of Medicine, University of Granada, 18071 Granada, Spain; 3 Biosanitary Institute of Granada (ibs.GRANADA), SAS-University of Granada, 18014 Granada, Spain; 4 Department of Microbiology, Science School, University of Granada, 18002 Granada, Spain; yjabalera@ugr.es

**Keywords:** Biomimetics, nanoparticles, colon carcinoma, liposome, Oxaliplatin

## Abstract

Current chemotherapy for colorectal cancer (CRC) includes the use of oxaliplatin (Oxa), a first-line cytotoxic drug which, in combination with irinotecan/5-fluorouracil or biologic agents, increases the survival rate of patients. However, the administration of this drug induces side effects that limit its application in patients, making it necessary to develop new tools for targeted chemotherapy. MamC-mediated biomimetic magnetic nanoparticles coupled with Oxa (Oxa-BMNPs) have been previously demonstrated to efficiently reduce the IC_50_ compared to that of soluble Oxa. However, their strong interaction with the macrophages revealed toxicity and possibility of aggregation. In this scenario, a further improvement of this nanoassembly was necessary. In the present study, Oxa-BMNPs nanoassemblies were enveloped in phosphatidylcholine unilamellar liposomes (both pegylated and non-pegylated). Our results demonstrate that the addition of both a lipid cover and further pegylation improves the biocompatibility and cellular uptake of the Oxa-BMNPs nanoassemblies without significantly reducing their cytotoxic activity in colon cancer cells. In particular, with the pegylated magnetoliposome nanoformulation (a) hemolysis was reduced from 5% to 2%, being now hematocompatibles, (b) red blood cell agglutination was reduced, (c) toxicity in white blood cells was eliminated. This study represents a truly stepforward in this area as describes the production of one of the very few existing nanoformulations that could be used for a local chemotherapy to treat CRC.

## 1. Introduction

Biominerals, especially those resulting from a biologically controlled mineralization process, have fascinated numerous researchers over the years due to their specific features and unique properties that are the result of the exquisite degree of control that the organisms have over the mineral formation [1]. Learning from nature and understanding how those complex biomineralization processes occur may provide insights for the design of new materials with improved properties. In this context, and taking inspiration from nature, novel magnetosome-like magnetite nanoparticles can be produced by means of magnetosome-associated proteins [2,3]. The main advantage of these magnetosome-like magnetic nanoparticles (namely biomimetic nanoparticles) versus magnetosomes is that their production could be scaled-up easily than that for magnetosomes, and their advantage over inorganic magnetic nanoparticles is that biomimetic ones show novel properties which make them promising nanocarriers [4,5,6].

To this extent, several magnetosome proteins, both full length proteins expressed as recombinant ones and synthetic peptides, have been tested in different in vitro magnetite precipitation experiments. Most of the work has been performed by using full length Mms6 from different *Magnetospirillum* species [7,8,9,10,11,12]. However, the advantages, in terms of drug nanocarriers of most of these nanoparticles compared to those chemically synthetized is not evident. MmsF from *Magnetospirillum magneticum* AMB-1 is another potential candidate for producing biomimetic nanoparticles [13]. However, these MmsF-mediated magnetic nanoparticles are still not characterized enough to determine whether or not they could be of use in nanotechnology. On the contrary, MamC-mediated biomimetic nanoparticles (here referred as BMNPs) have demonstrated their potential as promising drug nanocarriers [5,6,14,15] and as hyperthermia agents [16,17], opening the possibility of a combined therapy by using the same nanoplatform. MamC controls magnetite nucleation and growth by both template and ionotropic effects [18], remaining attached to the nanoparticles and forming a nanocomposite (95 wt% magnetite + 5 wt% MamC) that results, not only in magnetic nanoparticles of different size and morphology (and thus magnetic properties) compared to those of chemically produced ones, but also in nanoparticles with novel surface properties [5]. In fact, these BMNPs display larger sizes (~40 nm) compared to most commercial chemically synthesized magnetic nanoparticles (MNPs) (≤ 30 nm), and are endowed of (1) a higher blocking temperature while being superparamagnetic at room temperature and (2) high saturation magnetization, these features being consistent with well-structured magnetic nanoparticles with a large magnetic moment per particle. These characteristics (i) reduce aggregation of BMNPs due to magnetic dipole particle interaction in the absence of an external magnetic field, while (ii) increase the intensity of the BMNPs response to an external magnetic field, thus increasing the efficiency of their magnetic guidance. Moreover, in terms of surface properties, MamC confers new surface properties to these BMNPs, in particular, an isoelectric point (pI) at pH 4.4 which allows functionalization based on electrostatic interactions and drug release being pH dependent. In fact, a previous proof of concept showed that BMNPs were biocompatible and could be coupled with Oxaliplatin (Oxa) [14]. Therefore, these MamC-mediated BMNPs have been proposed as efficient nanocarriers [5,6,14,15] of a number of molecules offering crucial advantages over the use of inorganically synthesized magnetic nanoparticles to this end.

In this context, the potential application of BMNPs for the local and guided delivery of Oxa to treat colorectal cancer (CRC) stands out as a novel therapy. In fact, Oxa and 5-fluorouracil (5-FU) are drugs of choice on the advanced CRC (5-FU/Leucovorin, FOLFOX, CAPOX, FOLFIRINOX) [19,20], the fourth most frequent cancer and the second leading cause of cancer death [21]. Despite the effectiveness of these drugs, the many side effects linked to their systemic use (fatigue, neuropathy, diarrhea, acneiform dermatitis, nausea, neutropenia, anemia and hand-foot syndrome) [22,23] seriously limit the success of this treatment in CRC. In particular, Oxa induces peripheral sensory neurotoxicity [19] which may be the cause to discontinuation of drug treatment.

Given these facts, the design of a new strategy to locally deliver Oxa at the target site would represent a great stepforward in the treatment of CRC. Several attempts have been made in this regard with this and other cancer types. For instance, Harada et al. recently demonstrated a significant reduction of adverse effects (AEs) using a paclitaxel (PTX) nanoformulation (nab-PTX) in a phase I/II study of advanced non-small cell lung cancer (NSCLC) [24]. Fujiwara et al. showed a decrease (81.3%) of the peripheral sensory neuropathy with the use of polymeric micelles loaded with PTX (NK105) in patients with breast cancer (phase III) [25]. In addition, a camptothecin nanoformulation achieved a pathologic complete response with only a grade 4 toxicity event in patients with advanced rectal cancer (phase Ib/II study) [26]. Finally, NAPOLI-1 trial (phase III) assayed the administration of nal-IRI+5-FU/LV which increases the time without AEs ≥ 3 grade compared to 5-FU/LV in pancreatic cancer [27].

The use of our BMNPs as drug nanocarriers (Oxa) and as hyperthermia agents have the advantage over the abovementioned systems of: (1) the magnetic guidance allow both the control of where the drug is delivered and the concentration of the drug at the target thanks to possibility of a magnetic guidance and (2) the potentiation of the cytotoxic effect by using a combined therapy based on chemotherapy and magnetic hyperthermia [14]. Oxa–BMNP nanoassemblies increased the cytotoxic effect of the drug to even higher level than that caused by soluble Oxa, probably due to the internalization of BMNPs in the tested colon cancer cells.

However, as a drawback, there was a strong interaction of the nanoassemblies with the macrophages thus reducing the viability of these cells. Therefore, the system should be improved with the goal of an in vivo application. In the present study, and in order to reduce the interaction of the Oxa-BMNPs nanoassemblies with the blood cells, these nanoassemblies were enveloped in phosphatidylcholine unilamellar liposomes (both pegylated and non-pegylated) extruded to sizes of <100 nm and <200 nm. The stability, drug release, interaction with cell blood components and cytotoxicity in different colon cancer cells is here explored. It is true that magnetoliposomes containing synthetic MNPs have already been proposed to more or less extent, but they have the drawbacks exposed above linked to the relatively small magnetic moment per particle of most MNPs and the difficulties with the functionalization of the MNPs given their pI of ~7. Therefore, magnetoliposomes containing BMNPs are expected to perform better in terms of drug delivery and magnetic guidance. There are very few manuscripts regarding magnetoliposomes containing BMNPs. In fact, only magnetoliposomes containing Mms6-mediated BMNPs have been described so far, and their production is intended for magnetic resonance imaging [28,29,30]. Therefore, to our understanding, this is the first attempt to produce magnetoliposomes containing MamC-mediated BMNPs for a directed delivery of oxaliplatin, and it is also one of the first tryout for the local delivery of this drug, which may be of potential interest to locally treat CRC.

## 2. Materials and Methods

### 2.1. Preparation of Oxa-BMNP Nanoassemblies

Biomimetic magnetic nanoparticles (BMNPs) were synthetized by using the protocols described previously [4,31] by using the magnetosome membrane protein MamC (expressed as recombinant). Briefly, MamC protein was expressed in *Escherichia coli* TOP10 (Life Technologies: Invitrogen, Grand Island, NY, USA) and was purified by fast protein liquid chromatography (FPLC, GE Healthcare) by using immobilized metal affinity chromatography (IMAC, GE Healthcare, Chicago, IL, USA). The synthesis of BMNPs was carried out at 25 °C and 1 atm total pressure from oxygen-free solutions containing 3.5 mM NaHCO_3_, 2.78 mM Fe(ClO_4_)_2_, 3.5 mM Na_2_CO_3_, 5.56 mM FeCl_3_, and 10 µg/mL recombinant MamC, at a pH value of 9. All experiments were done under anoxic conditions inside an anaerobic Coy chamber (96% N_2_/4% H_2_). BMNPs were washed, sterilized and stored in HEPES buffer (pH 7.4) inside the Coy Chamber at 25 °C until further analyses. Identically, inorganic magnetic nanoparticles (MNPs) were produced under the same conditions but without adding MamC.

The functionalization of BMNPs with Oxaliplatin (Oxa) to form the nanoassembly Oxa–BMNPs was performed as detailed in [14]. Five milligrams of BMNPs were mixed with 1 mL of Oxa (2 mg/mL) in PBS buffer. Mixtures were incubated at 25 °C for 72 h. Then, the Oxa–BMNPs were magnetically collected and washed twice with 1 mL of PBS buffer. The amount of non-adsorbed Oxa was indirectly measured by ultraviolet–visible light (UV–Vis) spectroscopy at a wavelength of 240 nm in all the supernatant collected. The encapsulation efficiency was 80 ± 10%. The Oxa-BMNPs nanoassemblies used in the present study have been previously characterized in Jabalera et al. [14].

### 2.2. Preparation of BMLs and Oxa-BMLs

Liposomes loaded with MNPs, BMNPs or Oxa-BMNPs (here referred as IMLs, BMLs and Oxa-BMLs, respectively, see Table 1) were synthesized according to previously described method [17] (Table 1). Five milligrams of egg phosphatidylcholine (PC) (Avanti Polar Lipids, ref. 840051) were dissolved in 6 mL of chloroform, which was then removed by vacuum rotatory evaporation. Then, the thin film was hydrated with 10 mg of MNPs, BMNPs or Oxa-BMNPs suspended in PBS buffer. For the PEGylation of magnetoliposomes, 1,2-distearoyl-sn-glycerol-3-phosphoethanolamine-N [methoxy (poly-ethyleneglycol)-2000] (DSPE-PEG, Avanti Polar Lipids, ref. 880120), was further added (8% molar ratio) to the lipid mixture during lipid film preparation. The lipid/NP ratio was fixed to around 10,000 lipids per nanoparticle to minimize the amount of free particles [32,33]. Samples were further extruded via 200 and 100 nm polycarbonate filters (Whatman) to obtain a uniform magnetoliposome aqueous dispersion (Avanti Mini Extruder, Avanti Polar Lipids). The different nanoformulations produced are listed in Table 1.

### 2.3. Characterization of Magnetoliposomes

Transmission electron microscopy (TEM) analyses were performed with a LIBRA 120 PLUS Carl Zeiss SMT electron microscope (Germany) on ultrathin sections (50–70 nm) of the samples embedded in Embed 812 resin and deposited onto copper grids. For magnetoliposomes, drops were placed on copper grids with formvar film and they were stained using negative staining technique. In addition, electron energy loss spectroscopy (EELS) was used to characterize the presence of iron content inside the liposomes. The hydrodynamic particle size of the samples at pH 7 was measured by dynamic light scattering (DLS) using a Nano-ZS apparatus (Malvern Instruments, Worcestershire, UK). For the measurement of electrophoretic mobility, stock suspensions of BMLs and Oxa-BMLs were suspended in flasks containing oxygen-free NaClO_4_ 10 mM (final volume of 10 mL/flask), and the pH was adjusted to 7.4. Samples were sonicated for 2 min, and the electrophoretic mobility was immediately measured. ζ-potential values were calculated from these measurements by using Malvern Zetasizer software (Malvern Instruments, UK). All measurements, done in triplicate for each sample, were carried out at 25 °C using disposable plastic cuvettes. Hysteresis cycles were carried out by using a superconducting quantum interference device (SQUID) 5 T magnetometer (Quantum Design MPMS XL, San Diego, CA, USA).

### 2.4. Cell Culturing

The colon cancer cell lines T84, HT29, SW480, HCT15 and MC38; and the normal cell lines RAW 264.7 (murine macrophages) and CCD18 (colon fibroblast) were grown in Dulbecco’s Modified Eagle’s Medium (DMEM), supplemented with 10% fetal bovine serum (FBS) and 1% of penicillin-streptomycin (Sigma–Aldrich, Madrid, Spain). White blood cells were cultured in RPMI-1640 Medium supplemented with 10% FBS and 1% of penicillin-streptomycin. All the cell lines were maintained at 37 °C in an atmosphere containing 5% CO_2_.

### 2.5. Blood Cells Compatibility of Liposomes and Magnetoliposomes

#### 2.5.1. Red Blood Cells Assay

Human blood from a healthy donor was obtained from Andalusian Biomedical Research Ethics Committee, Conserjería de Salud de la Junta de Andalucía, 2020522131049, PI19/01478 project, 25 November 2019). The potential hemolytic effect of LIP, IMLs and BMLs (pegylated or not) on red blood cells were studied by a previously established protocol [14]. Nanoformulation were added from stocks of different initial concentrations (10 µL of each) to allow a final NPs concentration in the wells ranging from 1 to 250 µg/mL. For positive and negative controls, 10 µL of 20%Triton X-100 and PBS pH 7.4 were used respectively. The samples were incubated at 37 °C under stirring for 1 h, centrifuged and 100 µL of the supernatant from each well were recovered, placed in a new flat-bottomed 96-well plate and measured at 492 nm (Titertek multiscan colorimeter, Flow, Irvine, CA, USA). The percentage of hemolysis (%HR) was calculated with this formula:

$$\%HR=\frac{Abs\;\left(sample\right)-Abs\;\left(-control\right)}{Abs\;\left(+control\right)-Abs\;\left(-control\right)}\times 100$$

After treatment, photographs of the samples were taken by light microscope (Leica DM IL LED).

#### 2.5.2. White Blood Cells Proliferation Assay

The effect of nanoformulations on white blood cells (WBC) was assessed using a formerly described protocol [14]. Briefly, WBCs were isolated from samples from healthy donors, cultured in 96-well plates at a density of 2 × 10^4^ cells/well in a volume of 90 µL and to which 10 µL of LIP, IMLs and BMLs (pegylated or not) were added to each well from stock solutions of different concentration to reach a final concentration of 1 to 250 µg/mL of Fe. The treatments were incubated for 1 and 12 h at 37 °C and 5% CO_2_ in a humidified atmosphere, after which the viability of the WBCs was determined by the Cell Counting Kit-8 (CCK-8) (Dojindo Laboratories, Kumamoto, Japan).

#### 2.5.3. Cell Cytotoxicity in RAW 264.7

Macrophages cell line RAW 264.7 was used to evaluate the toxicity of LIPs, IMLs and BMLs (pegylated or not). Cells were seeded in a 96-well plate at a density of 7.500 cell/well in a volume of 90 µL and treated with NPs (in 10 µL) to a range of different concentrations for 1 and 12 h. Then, cell viability was determined by adding MTT reagent (10%). After 4 h of incubation, the medium was discarded and a mixture of DMSO plus Sorensen buffer (8:1) was added to each well to resuspend the MTT crystals. Finally, the content of each well was recovered, placed in a new flat-bottomed 96-well plate and absorbance was measured at 570 nm (Titertek multiscan colorimeter Flow, Irvine, CA, USA).

### 2.6. In Vitro Proliferation Assays

Cells were seeded in 48-well plates (5 × 10^3^ cells/well for T84, CCD18 and HCT15, 1.5 × 10^4^ cells/well for HT29, 4 × 10^3^ cells/well for SW480 and 3 × 10^3^ cells/well for MC38,) and incubated overnight. Then, the different treatments, LIP, LIP-PEG, IMLs, IMLs-PEG, BMLs and BMLs-PEG, FeCl_3_ (control experiment)_,_ Oxa-BMLs, OXA-BMLs-PEG and soluble Oxa, were administered in a growing drug dose range. All nanoformulations were tested at both 100 and 200 nm. The viability was determined after 72 h by a Sulforhodamine B (SRB) as described previously [34].

### 2.7. Cell Uptake and Intracellular Location of BMLs and IMLs

#### 2.7.1. Cell Migration Assay

To assess the ability of IMLs, IMLs-PEG, BMLs and BMLs-PEG to migrate after being internalized in the presence of a magnetic field, cell lines were seeded in 6-well plates at a density of 3 × 10^5^ cells/well and exposed to 10 and 100 µg/mL of the different nanoformulations, BMNPs and FeCl_3_ at the equivalent iron concentration for 24 h. After that, the samples were processed and visualized as described previously [14].

#### 2.7.2. Cell Staining for Iron Determination

To observe the presence of BMLs and IMLs into tumor cells, T84 and RAW 264.7 cells were seeded on 12-well plates at a density of 2 × 10^5^ cells/well and incubated overnight. BMLs, IMLs, BMNPs and FeCl_3_ were added to the cell cultures from 10 to 100 µg/mL final iron concentration in culture medium. After 24 h, the cells were visualized by Prussian blue staining for iron detection as described previously [35] and samples were observed with a light microscope (Leica DM IL LED).

#### 2.7.3. Transmission Electron Microscopy Assays

To confirm the internalization of BMLs into tumor cells, SW480 and CCD18 cells were treated with BMLs 100 nm and BMLs-PEG 100 nm for 15 min, 2 and 24 h at a final iron concentration in culture medium of 10 µg/mL. Then, samples were processed at the Scientific Instrumentation Center (CIC) of the University of Granada as previously described [35] to transmission electron microscopy (TEM) observation. The ultrathin sections were examined in a LIBRA 120 PLUS (Carl Zeiss SMT, Jena, Germany) electron microscope.

### 2.8. Statistical Analysis

Statistical analysis was performed by using the Student’s t-test with the Statistical Package for the Social Sciences (SPSS) v.20. All the results were represented as mean ± standard deviation (SD). Data with *p* < 0.05 were considered as statistically significant.

## 3. Results

### 3.1. Characterization of the Nanoformulations

**Figure 1:**
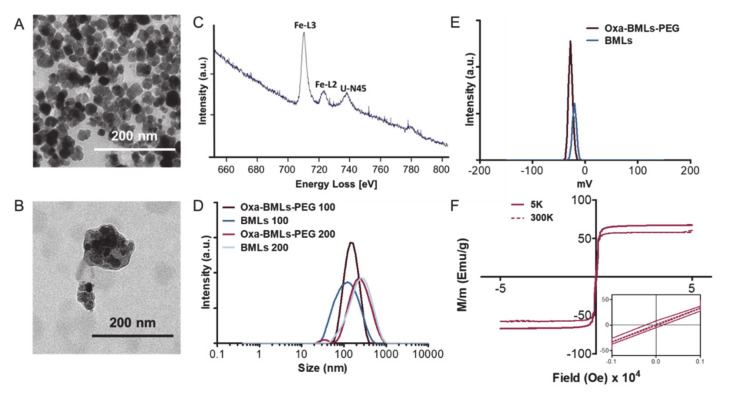
Characterizations of the different magnetoliposomes formulation. TEM images of (**A**) BMNPs and (**B**) Oxa-BMLs-PEG. (**C**) EELS spectrum of magnetoliposomes (U-N45 peak corresponds to uranile from negative staining). (**D**) Size distributions and (**E**) ζ potential of BMLs and Oxa-BMLs-PEG. (**F**) Hysteresis cycle of Oxa-BMLs-PEG.

Magnetic nanoparticles carrying oxaliplatin (Oxa-BMNPs) were entrapped inside the liposome, as demonstrated by TEM and EELS analyses (Figure 1A–C). In fact, EELS show the presence of iron, as expected for magnetite, inside the defined layer observed by TEM. BMNPs (Figure 1A) were uniform well-defined nanoparticles with a diameter of approximately 30–40 nm, while TEM analyses on Oxa-BMLs-PEG show particles with a diameter of 100–200 nm, enclosing several magnetic nanoparticles per liposome (Figure 1B). Identically, MNPs showed an average size value of 20 nm, while that for IMLs and IMLs-PEG was of 100 and 200 nm (Figure S1). Since BMNPs showed much better performance as Oxa carriers (detailed below), MNPs-bearing nanoformulations were not further used for the present study.

Dynamic light scattering (DLS) analysis show that the hydrodynamic diameters (Table S1) of the BMLs and Oxa-BMLs-PEG passed through a 100 nm extruder were of 98 ± 2 nm and 134.1 ± 0.9 nm, respectively and of 227 ± 4 nm and 235 ± 5 nm, respectively for BMLs and Oxa-BMLs-PEG passed through a 200 nm extruder (Figure 1D). The polydispersity index (PDI) of these samples were 0.253 ± 0.009, 0.162 ± 0.004, 0.38 ± 0.01 and 0.36 ± 0.02, respectively, which indicates the good dispersity of the samples. In all cases, the size of the Oxa-BMLs-PEG was larger than that of the BMLs due to the presence of both the oxaliplatin coating on the BMNPs and the PEG group at the liposomes surface.

Both BMLs and Oxa-BMLs-PEG are negatively charged at pH 7.4, (ζ potential of −20.4 ± 0.9 mV and –27.3 ± 0.4, respectively, Figure 1E and Table S1). These negative charge in also observed for other liposome involving compositions [35,36,37] and it has been attributed to the coverage with OH^−^ of the –N(^+^)(CH_3_)_3_ group of the phosphatidylcholine [38]. The fact that these nanoformulations are negatively charged may facilitate the colloidal stability of the suspension by means of electrostatic repulsion.

The hysteresis loop of Oxa-MBLs-PEG showed a typical ferromagnetic behavior at 5 K, while at 300 K, these formulations showed zero coercivity, which indicates their superparamagnetic character, being the magnetization saturation (Ms) at 300 K 60 emu/g (Figure 1F).

### 3.2. BMLs and IMLs Biocompatibility in Blood Cells

**Figure 2:**
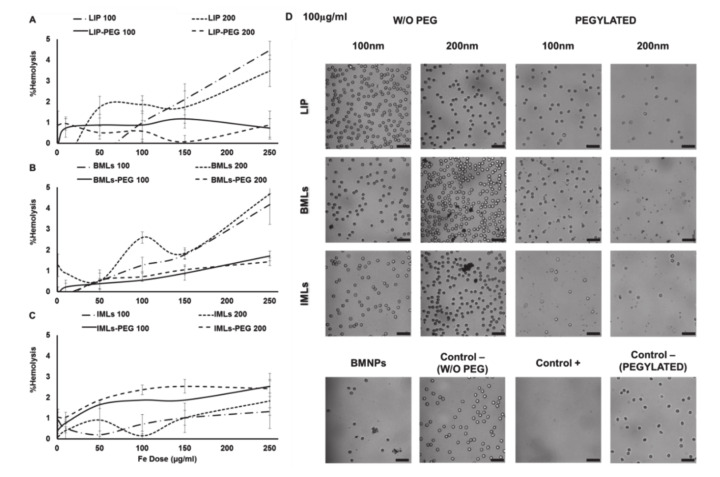
LIP, BMLs and IMLs biocompatibility with red blood cells. Hemolysis test with (**A**) LIP, (**B**) BMLs and (**C**) IMLs. Data represent the mean values ± SD of triplicate samples. (**D**) Images of optical microscopy of erythrocytes after treatment with the different nanoformulations at 100 µg/mL of Fe. Scale bar 25 µm.

As for the hemolysis test, LIP and BMLs of both sizes are the most hemolytic nanoformulations (~5% hemolysis), however, with pegylation, their percentage of hemolysis decreases to ~1% and <2% (respectively), being then considered biocompatible (Figure 2A,B) [39]. On the contrary, pegylation increases the hemolysis percentage from ~1% to ~2% in IMLs (Figure 2C). It is worth noticing that the treatment with pegylated nanoformulations, even at the highest doses (250 μg/mL, images not shown), eliminate the agglutination of the erythrocytes (Figure 2) previously observed with BMNPs at lower doses (Figure 2D).

**Figure 3:**
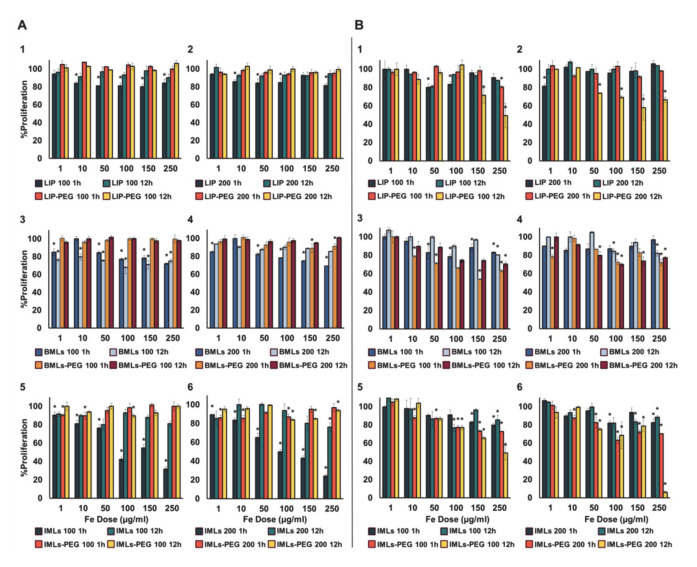
Toxicity test in human white blood cells (WBC) (**A**) and RAW 264.7 macrophages (**B**). Data represent the mean values ± SD of triplicate samples. Statistically significant differences with respect to untreated control (*p* < 0.05) are represented with *.

The administration of non-pegylated IML nanoformulations induces a dose-dependent toxicity in WBCs at 1 h of treatment (Figure 3(A5),(A6)), which becomes more noticeable as the size of the magnetoliposome increases. In contrast, LIP (Figure 3(A1),(A2)) and BMLs (Figure 3(A3)) induce a ~ 20% toxicity regardless of the dose (with the exception of 200 nm BMLs in which a dose dependent toxicity is observed; Figure 3(A4)). At 12 h exposure treatments an almost total recovery of the WBCs occurred, with the exception of 100 nm BMLs (Figure 3(A3)). Different scenario is the case of treatments with pegylated nanoformulations, in which no toxicity is observed for WBC at any of the doses studied, in any of the nanoformulations tested, both at 1 h and at 12 h of exposure.

In the context of toxicity for RAW 264.7 cell line (Figure 3B), our results demonstrate that the pegylated nanoformulations are more toxic than non-pegylated ones, being that especially true for IMLs. This toxicity increases while increasing magnetoliposome size and exposure time (Figure 3(B5),(B6)).

**Figure 4:**
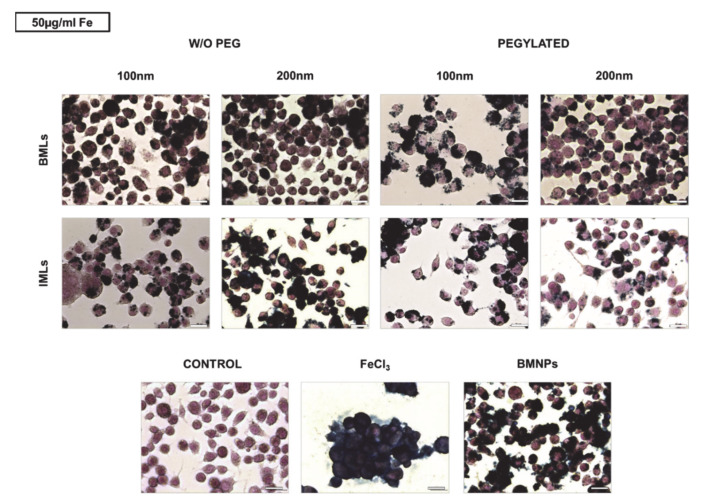
Internalization of BMLs and IMLs in RAW 264.7 cells. Representative images of RAW 264.7 cells exposed to 50 µg/mL Fe of BMLs and IMLs (pegylated or not), BMNP (magnetic core) and Fe for 24 h and stained with Prussian blue. Scale bar 25 µm.

Prussian Blue staining was performed to qualitatively check the internalization of the nanoformulations with magnetic core in the RAW 264.7 cell line. To this purpose, doses of 10, 50 and 100 μg/mL Fe of the different nanoformulations were administered for 24 h. Figure 4 shows representative images of the treatments at doses of 50 μg/mL. It can be seen that, while macrophages are capable of accumulating large amounts of Fe in solution (treatment with FeCl_3_), the administration of BMNPs reduces such Fe internalization, because the nanoformulations adhere to the surface and only a small proportion is internalized. Internalization increases in magnetoliposomes compared with uncovered nanoparticles, being such internalization higher in non-pegylated versus pegylated nanoformulations and in BMLs versus IMLs. In addition, internalization is favored as the size of the magnetoliposome increases.

### 3.3. In Vitro Proliferation Assays

**Figure 5:**
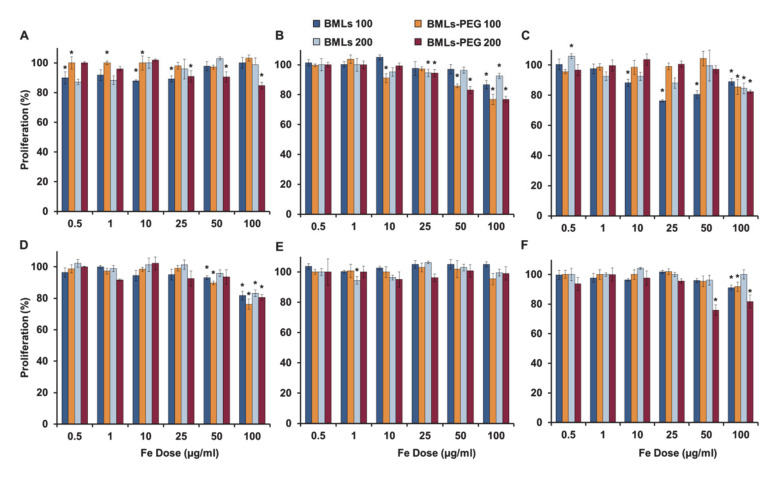
Cell proliferation assay of colon cell lines treated with BMLs and BMLs-PEG. The cell lines T84 (**A**), CCD18 (**B**), SW480 (**C**), HCT15 (**D**), HT29 (**E**) and MC38 (**F**) were exposed to increasing concentrations of Fe from 0.5 to 100 µg/mL from BMLs and BMLs-PEG for 72 h. The graphs represent the percentages of proliferation of all the cell lines obtained using the SRB assay. Data represent the mean values ± SD of triplicate cultures. Statistically significant differences with respect to untreated control (*p* < 0.05) are represented with *.

Regarding the biocompatibility of the nanoformulations, a great cytocompatibility of the BMLs (pegylated and non-pegylated) was observed (Figure 5). In HT29, no toxicity is observed at any of the tested doses, while in HCT15, CCD18 and MC38 cell lines only slight toxicity occurs at the higher doses. In MC38, only a significant toxicity is observed when treated with BMLs-PEG 200 nm at the higher doses while in CCD18 the pegylation produces a higher toxicity at high doses. On the other hand, in SW480 cell line, pegylation improves cytocompatibility at low doses (Figure 5C).

As for the rest of nanoformulations (non-pegylated, Figure S2 and pegylated, Figure S3), it can be observed that non-pegylated LIP present toxicity in the SW480, HCT15 and MC38 lines in a dose-dependent manner, being higher the toxicity in the MC38 cell line. However, pegylation solves this toxicity. The administration of free iron (FeCl_3_) produces toxicity in the T84, CCD18, SW480, HT29 and MC38 cell lines, especially at high doses; however, the administration of magnetite nanoformulations at the same doses of iron does not match in any case this toxicity except for the CCD18 cell line that is especially sensitive to iron administration. In CCD18 the administration of IMLs-PEG was much more toxic than the administration of IMLs. In addition, an absence of toxicity was observed in the administration of BMLs and BMLs-PEG in this cell line. On the other hand, in MC38, the administration of IMLs-PEG at high doses is toxic, however this toxicity is lower than that observed with the administration of free iron. In addition, as is also observed in the administration of BMLs-PEG, the size of 200 nm is more toxic in this line. In general, there are no significant differences between the different sizes of the different nanoformulations.

**Figure 6:**
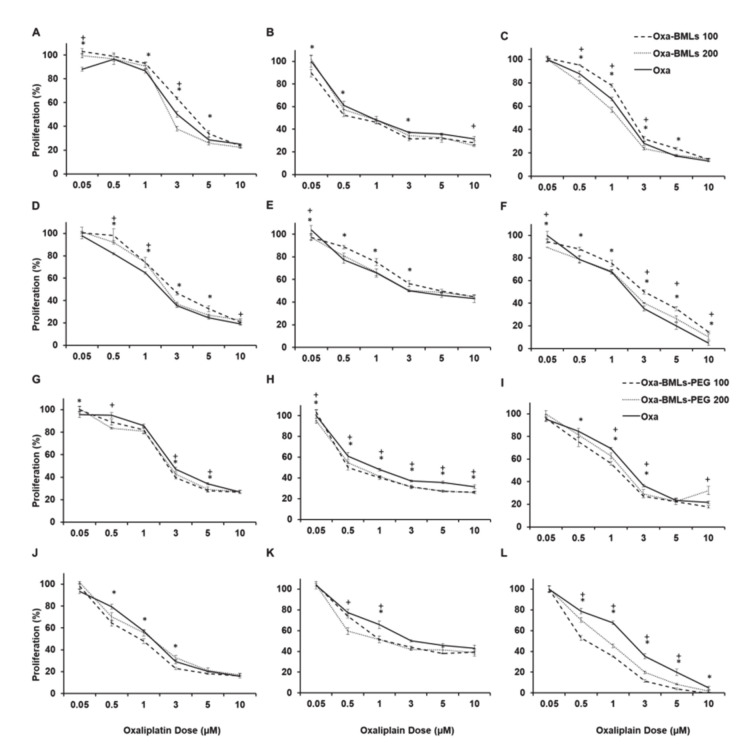
Cell proliferation assay of colon (tumor and non-tumor) cell lines treated with soluble Oxa, Oxa–BMLs (**A**–**F**) and Oxa–BMLs-PEG (**G**–**L**). The percentages of proliferation of T84 (**A**,**G**), CCD18 (**B**,**H**), SW480 (**C**,**I**), HCT15 (**D**,**J**), HT29 (**E**,**K**) and MC38 (**F**,**L**) cell lines obtained using are shown. Data represent the mean values ± SD of triplicate cultures. Statistically significant differences with respect to free Oxa (*p* < 0.05) are represented with * for NPs of 100 nm and with + for NPs of 200 nm.

All treatments with Oxa-BMLs (100 and 200 nm), pegylated or not (Figure 6), have a similar or higher effect to the administration of soluble Oxa, improving in some cases its effect. In all cases there are significant differences (*p* < 0.05) between the IC_50_ of the treatments in the different lines except for CCD18 (all the treatments), Oxa-BMLs 200 nm in HT29 and the Oxa-BMLs-PEG 200 nm in SW480. In HCT15 there are no significant differences between the IC_50_ of the BMLs-PEG with the IC_50_ of soluble Oxa. The IC_50_ of the different treatments are shown in Table 2.

### 3.4. Cell Migration under a Magnetic Field In Vitro

**Figure 7:**
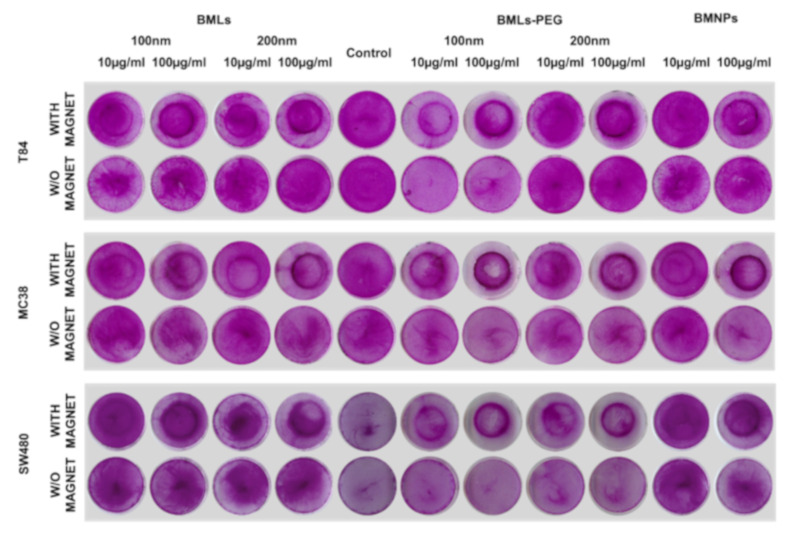
Cell migration assay. Representative image of colon cancer cells (T84, MC38 and SW480) exposed to different concentrations of BMLs, BMNPs and FeCl_3_ and stained with SRB. Migration of the cells after treatments was evaluated in the presence or absence of a magnet.

BMLs (Figure 7) and IMLs (Figure S4) promote magnetic migration when the petri dish containing the cells is exposed to a magnet. Such a magnetic migration occurs to a greater extent in liposome-bearing compared to uncoated ones. In addition, pegylation seems to enhance this migration even further. In fact, when looking closely to the dark halo resulting from the magnet treatment in non-pegylated nanoformulations, it can be concluded that it is created by non-internalized ones, which does not occur in pegylated nanoformulations. This observation was more noticeable in IMLs (all cell lines at doses of 100 μg/mL) than in BMLs nanoformulations (MC38, BMLs 200 nm, 100 μg/mL).

### 3.5. BMLs Internalization in Colon Cell Lines

**Figure 8:**
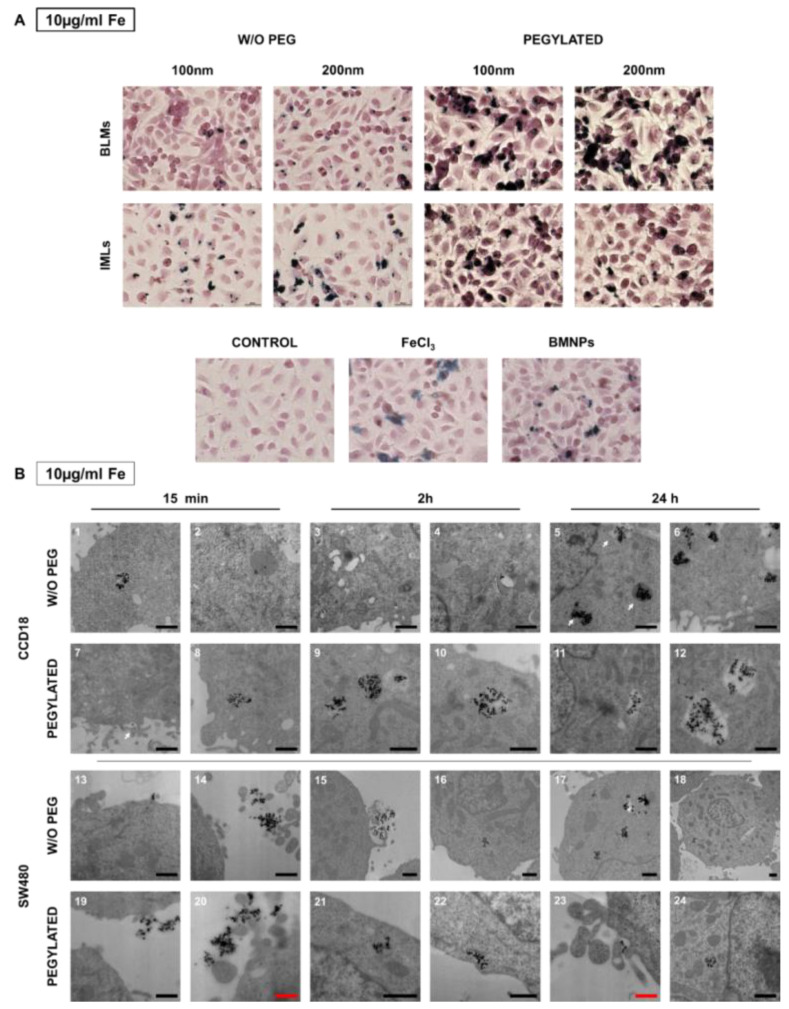
Internalization of BMLs and IMLs in colon cancer cells. (**A**) Representative images of T84 human cancer cells exposed to 10 µg/mL Fe of BMLs and IMLs (pegylated or not), BMNP (magnetic core) and Fe for 24 h and stained with Prussian blue. Scale bar 25 µm. (**B**) Transmission electron microscopy analysis of the BMLs 100 nm internalization in colon cell lines. Representative images of CCD18 cell line (1–12) and SW480 colon tumor cells (13–24) showing different states of internalization of BMLs and BMLs-PEG, 100 nm both, at 10 µg/mL Fe. Black scale bar, 1 µm and red scale bar 0.5 µm.

To evaluate the cellular uptake of the nanoformulations, Prussian Blue staining was performed after administration of the nanoformulations at 10, 50 and 100 μg/mL. Figure 8A shows representative images of the 10 μg/mL dose, since at lower doses a differential behavior of the different nanoformulations is observed that is not clearly identified at higher doses. Opposite to what was observed for macrophages, pegylation promotes the internalization of the nanoformulations in T84. This event can be observed more noticeably for BMLs than for IMLs, and for the 200 nm size.

In CCD18 internalization of pegylated and non-pegylated nanoformulations was observed even at short exposures (15 min). At 24 h large vesicles full of nanoformulations could be detected. The suggested process sequence could be identified as follows (Figure 8(B5)): a vesicle containing the nanoformulations (middle arrow), a digestive vesicle (upper arrow) and the nanoformulations that are released into the cytoplasm (lower arrow). Such a fast internalization (15 min) does not seem to occur in SW480 (Figure 8(B13–14) and Figure 8(B19–20)). In fact, BMLs-PEG accumulations start being observed inside the cell after 2 h of exposure and continue at 24 h.

## 4. Discussion

**Figure 9:**
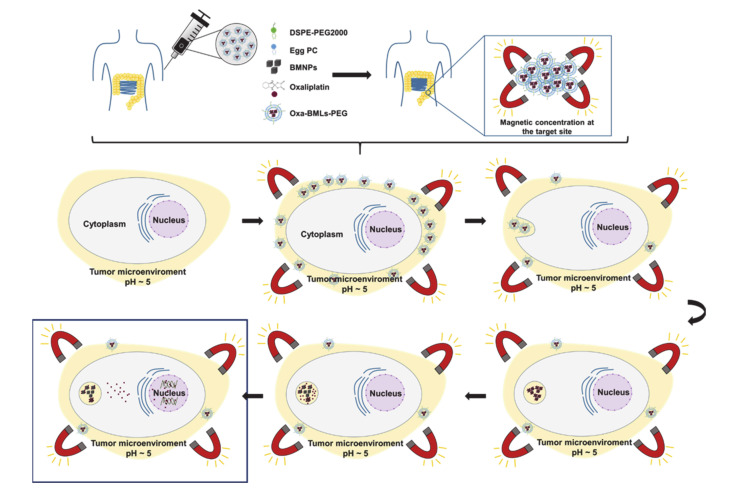
Proposed model of the different mechanisms of action of Oxa-BMLs-PEG in cancer cells.

Our model for the functioning of the magnetoliposome is illustrated in Figure 9. After intravenous injection (or direct injection at the tumor site) and magnetic concentration at the target site, the magnetoliposomes could release their content by different mechanisms: (a) magnetoliposomes could interact with the plasma membrane and discharge the nanoassemblies into the cytoplasm, (b) the interaction with the cell could alter the structure of the bilayer and release the nanoassemblies [40], and (c) magnetoliposomes could enter the cell by endocytosis (probably clathrin-mediated endocytosis) and then, the disruption of the lipid cover could occur by merging with the endosomes membranes [40,41,42]. It has also been shown that the lipid cover can be broken by applying magnetic hyperthermia [17]. Once liberated, Oxa-BMNPs nanoassemblies would be enclosed in endosomal/lysosomal compartments at a pH value of 4–5 [43,44,45,46]. At this acidic pH value, the lipid membrane is degraded [40] and the BMNPs becomes nearly uncharged (pI = 4.4, [5]), releasing the drug that was previously attached by electrostatic interaction [14]. Such an Oxa release could be further increased if magnetic hyperthermia is applied [14]. The released Oxa diffuses toward the cytoplasm and a percentage enters the cell nuclei, as previously shown to occur for doxorubicin when administered as DOXO-BMNPs nanoassemblies [6].

In comparison to our previous studies [14], the addition of a lipid cover to the nanoparticles improve their dispersion, eliminating the agglutination of erythrocytes previously observed. In addition, the pegylation of both LIP and BMLs reduced their blood toxicity to values considered biocompatible (<2%) [39]. Only IML pegylation induced a slight increase on their hemolysis capacity (1% to ~2%), although within the normal range of hemolysis that is considered biocompatible (0–2%) [47]. Our results are in agreement with the findings of other authors, who documented that pegylation reduced blood proteins binding [48] such as BSA and IgG [49] improving the dispersibility of the nanoparticles and reducing their interactions [50]. On the other hand, pegylation also significantly reduce the interaction between the nanoformulations and the reticuloendothelial system cells such as lymphocytes and macrophages [49,51]. In this context, firstly, our results demonstrated that BMLs-PEG were non-toxic to WBC both at 1 and 12 h of exposure by contrast to uncovered NPs in addition to observing a recovery of viability in most treatments after 12 h of exposure such as has been previously described [14]. Secondly, a significant toxicity decrease (40%) of the BMLs-PEG was observed in macrophages in relation to previously obtained data of uncoated BMNPs in the same type of cells. This fact may be due to the lower internalization of the pegylated nanoformulations (Figure 4) also in agreement with the finding that pegylation produces a lower internalization of iron oxide nanoparticles in the RAW 267.4 cell line [49,51,52]. The pegylated nanoplatforms (LIP-PEG, BMLs-PEG and IMLs-PEG) were slightly more toxic in macrophages versus the non-pegylated (LIP, BMLs and IMLs) although we cannot explain it. In fact, it is remarkable the differential behavior of LIP, BMLs and IMLs in this cell line. While LIPs and BMLs show a slight toxicity at very high doses (250 µg/mL) at 12 h of exposure, IMLs show a dramatic reduction of viability. In this context, both magnetic nanoplatforms, BMLs and IMLs, have the same lipid cover, the same functionalization (PEG) and a core composed by Fe_3_O_4_. However, BMLs have a biomimetic core with the MamC protein strongly adhered to its surface [5] while IMLs have a core with a free surface. It is widely reported in the literature that the accessibility of chelating agents that degrade iron oxide crystal depends on its functionalization and that this degradation begins first on surfaces where functionalization is less dense [53,54]. Furthermore, the fact that large concentrations of IONPs produce high levels of oxidative stress in the RAW 267.4 cell line [55,56] lead us to suggest that IMLs could degrade more rapidly inside the cells than BMLs causing greater toxicity. For this reason, it is important to note that the presence of the MamC protein on the surface of the core of BMLs not only slows down the degradation of the core, making the release of iron ions more gradual and therefore less toxic, but also increases their useful life allowing that they can be used for hyperthermia at longer exposure times without repeating their inoculation. Finally, the lower internalization of BMLs compared to uncovered BMNPs [14] can also be related with the great reduction of toxicity observed in the normal CCD18 versus previous studies [14].

In the context of the Oxa-BMNPs nanoassemblies, the addition of both a lipid shell and further pegylation not only improves the biocompatibility of the nanoassembly, but also the antiproliferative effect compared to that of soluble OXA. A reduction in the IC_50_ of free OXA is observed in most tumor cell lines when treated with pegylated nanoformulations, being especially remarkable such reduction in HT29. The reduction of the IC_50_ with the new nanoformulations (Oxa-BMLs and Oxa-BMLs-PEG) is not as pronounced as it was with the Oxa-BMNPs nanoassemblies of previous works described by Jabalera et al. [14], which is probably due to the lower internalization of the former, as previously described above and in accordance with results from other authors [57,58]. Results from this study represent a true stepforward in this research area, since it is the first study in which nanoformulations are designed to locally release Oxa in which both a reduction of the IC_50_ compared to that for free Oxa, and an improvement of their biocompatibility and cellular uptake have been demonstrated. Other studies in which an Oxa directed chemotherapy was also intended [59,60] have failed in demonstrating such an improvement in the interaction with blood or normal tissue cells for their formulations (OXA-DACHPt-loaded nanoparticles and IOHNPs), being that essential for a potential application.

Internalization and migration assays of BMLs showed an increase of nanoformulations inside the cells. Therefore, our results clearly show that the addition of both, lipid envelope and pegylation, to our nanoassemblies (Oxa-BMLs) or to the biomimetic nanoparticles (BMLs) increased their cell uptake compared to that of raw Oxa-BMNPs and/or BMNPs. In terms of cell internalization, it is well documented that SPIONs can accumulate in large amounts inside the cell, both in cancer cells and in normal cells [61,62]. Our hypothesis is that the coating and functionalization of the nanoformulations increase their hydrophilicity and therefore their dispersion [63], and reduce the interactions between particles [50], allowing a greater number of nanoformulations to be available for cell internalization, which previously remained agglutinated. A greater amount of NPs (and thus, also iron) inside the cell increases migration capacity. In any case, our results demonstrated that our internalized nanoformulations maintained their magnetic properties, which could be used in different therapeutic strategies such as the application of a magnetic field to induce hyperthermia.

## 5. Conclusions

The results from the present study demonstrate that biomimetic MamC-mediated nanoparticles are one of the few Oxa nanocarriers with a significant potential for a directed local chemotherapy against CRC. The strong interaction of the nanoassembly Oxa-BMNPs with the macrophages is overcome if this nanoassembly is enveloped in a liposome that is further pegylated. The study of the biocompatibility in blood cells, cell internalization capability and cytotoxicity assays performed in the present work show that the enveloping in a lipid shell and the further pegylation improves the Oxa-BMNPs biocompatibility and cellular uptake without significantly reducing the Oxa cytotoxic activity in colon cancer cell lines. The present study is a crucial proof of concept necessary for the future potential application of these novel nanoformulations.

## Figures and Tables

**Table 1:** Synthetized nanoformulations.

Abbreviation	Composition
LIP	Empty liposome (without magnetite)
LIP-PEG	Empty pegylated liposome (without magnetite)
IMLs	Magnetoliposome containing MNPs
IMLs-PEG	Pegylated magnetoliposome containing MNPs
BMLs	Magnetoliposome containing BMNPs
BMLs-PEG	Pegylated magnetoliposome containing BMNPs
Oxa-BMLs	Magnetoliposome containing BMNPs loaded with oxaliplatin (Oxa-BMNPs)
Oxa-BMLs-PEG	Pegylated magnetoliposome containing Oxa-BMNPs
BMNPs	Biomimetic magnetic nanoparticles
All liposomes and magnetoliposomes were synthesized at 100 and 200 nm. BMNPs had an average size of 34 nm.	

**Table 2:** Oxa-BMLs and Oxa-BMLs-PEG IC_50_ (µM) in colon cell lines.

Cell Line	OXA	Oxa-BMLs		Oxa-BMLs-PEG	
		100 nm	200 nm	100 nm	200 nm
**T84**	3.21 ± 0.21	3.95 ± 0.25	2.71 ± 0.29	2.60 ± 0.33	2.71 ± 0.27
**CCD18**	1.09 ± 0.17	0.87 ± 0.16	1.20 ± 0.25	0.81 ± 0.22	0.84 ± 0.15
**SW480**	1.81 ± 0.12	2.15 ± 0.16	1.35 ± 0.10	1.36 ± 0.12	2.14 ± 0.46
**HCT15**	1.60 ± 0.09	2.80 ± 0.23	2.31 ± 0.22	0.96 ± 0.09	1.33 ± 0.10
**HT29**	4.02 ± 0.74	5.57 ± 0.63	4.64 ± 0.72	2.54 ± 0.68	2.19 ± 0.75
**MC38**	1.67 ± 0.12	2.83 ± 0.17	1.88 ± 0.17	0.60 ± 0.04	0.93 ± 0.04

Data represent the mean values ± SD of triplicate cultures.

## Supplementary Materials

The following are available online at https://www.mdpi.com/1999-4923/12/6/589/s1. Figure S1: Characterizations of the different formulations. TEM images of (A) LIPs, (B) IMLs and (C) BMNPs. (D) Size distributions and (E) mean of LIPs and IMLs, Figure S2: Cell proliferation assay of colon cell lines treated with FeCl3, LIP and IMLs. The cell lines T84 (A), CCD18 (B), SW480 (C), HCT15 (D), HT29 (E) and MC38 (F) were exposed to increasing concentrations of Fe from 0.5 to 100 µg/mL from the different NPs for 72 h. The graphs represent the percentages of proliferation of all the cell lines obtained using the SRB assay. Data represent the mean values ± SD of triplicate cultures, Figure S3: Cell proliferation assay of colon cell lines treated with FeCl_3_, LIP-PEG and IMLs-PEG. The cell lines T84 (A), CCD18 (B), SW480 (C), HCT15 (D), HT29 (E) and MC38 (F) were exposed to increasing concentrations of Fe from 0.5 to 100 µg/mL from the different NPs for 72 h. The graphs represent the percentages of proliferation of all the cell lines obtained using the SRB assay. Data represent the mean values ± SD of triplicate cultures, Figure S4: Cell migration assay. Representative image of colon cancer cells (T84, MC38 and SW480) exposed to different concentrations of IMLs, BMNPs and FeCl_3_ and stained with SRB. Migration of the cells after treatments was evaluated in the presence or absence of a magnet, Table S1: Average size and potential zeta values of different magnetoliposomes formulations.
