# Supplementary material for: Biomimetic Magnetoliposomes as Oxaliplatin Nanocarriers: In Vitro Study for Potential Application in Colon Cancer
Source: Pharmaceutics. 2020 Jun 24;12(6):589. doi: 10.3390/pharmaceutics12060589 (PMC7356838; doi:10.3390/pharmaceutics12060589)
Supplement: Supplementary file 1 [file pharmaceutics-12-00589-s001.pdf]

pharmaceutics

Supplementary material

Biomimetic Magnetoliposomes as Oxaliplatin Nanocarriers: *In Vitro* Study for Potential Application in Colon Cancer

Beatriz Garcia-Pinel[a,b,c,†], Ylenia Jabalera[d,†], Raul Ortiz[a,b,c], Laura Cabeza[a,b,c,] , Concepcion Jimenez-Lopez[d,*], Consolación Melguizo[a,b,c,*] and José Prados[a,b,c,*]

[1] Institute of Biopathology and Regenerative Medicine (IBIMER), Center of Biomedical Research (CIBM), University of Granada, 18100 Granada, Spain.
[2] Department of Anatomy and Embriology, Faculty of Medicine, University of Granada, 18071 Granada, Spain.
[3] Biosanitary Institute of Granada (ibs.GRANADA), SAS-University of Granada, 18014 Granada, Spain.
[4] Department of Microbiology, Science School, University of Granada, 18002 Granada, Spain

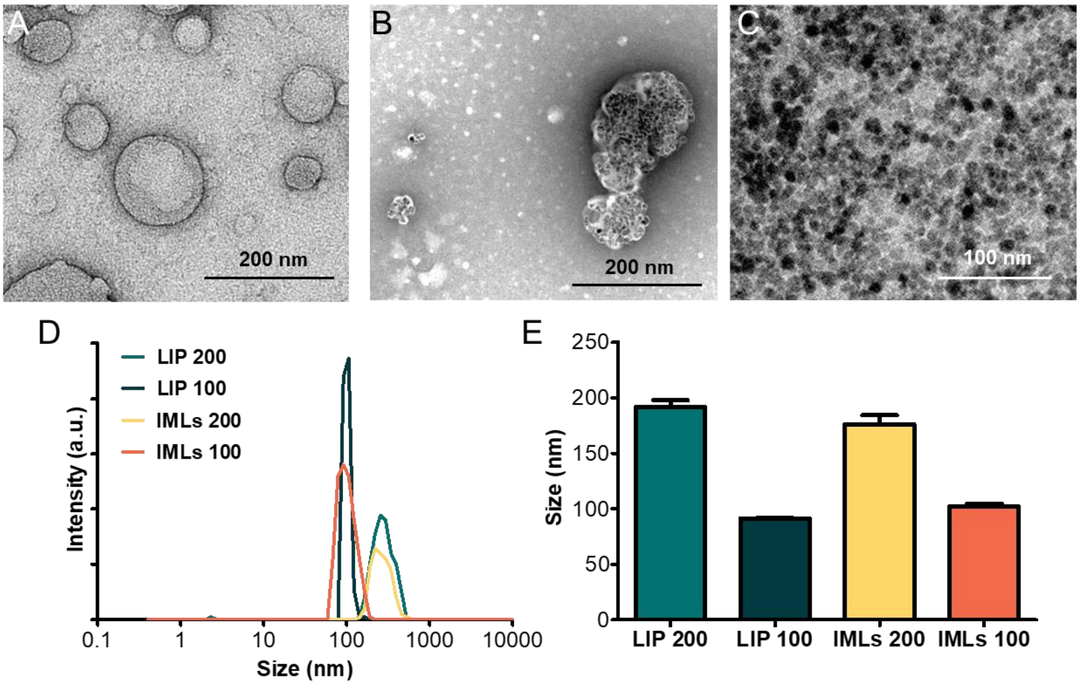

Figure S1. Characterizations of the different formulations. TEM images of (A) LIPs, (B) IMLs and (C) BMNPs. (D) Size distributions and (E) mean of LIPs and IMLs.

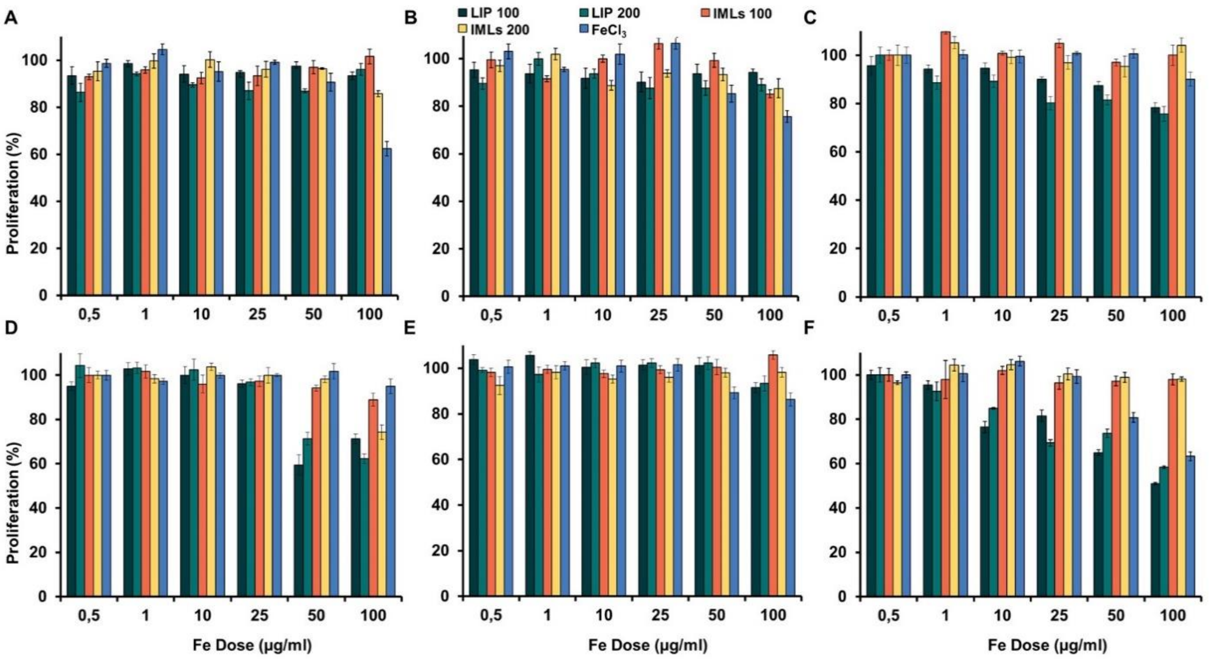

Figure S2. Cell proliferation assay of colon cell lines treated with $FeCl_3$, LIP and IMLs. The cell lines T84 (A), CCD18 (B), SW480 (C), HCT15 (D), HT29 (E) and MC38 (F) were exposed to increasing concentrations of Fe from 0.5 to 100 μg/mL from the different NPs for 72 h. The graphs represent the percentages of proliferation of all the cell lines obtained using the SRB assay. Data represent the mean values ± SD of triplicate cultures.

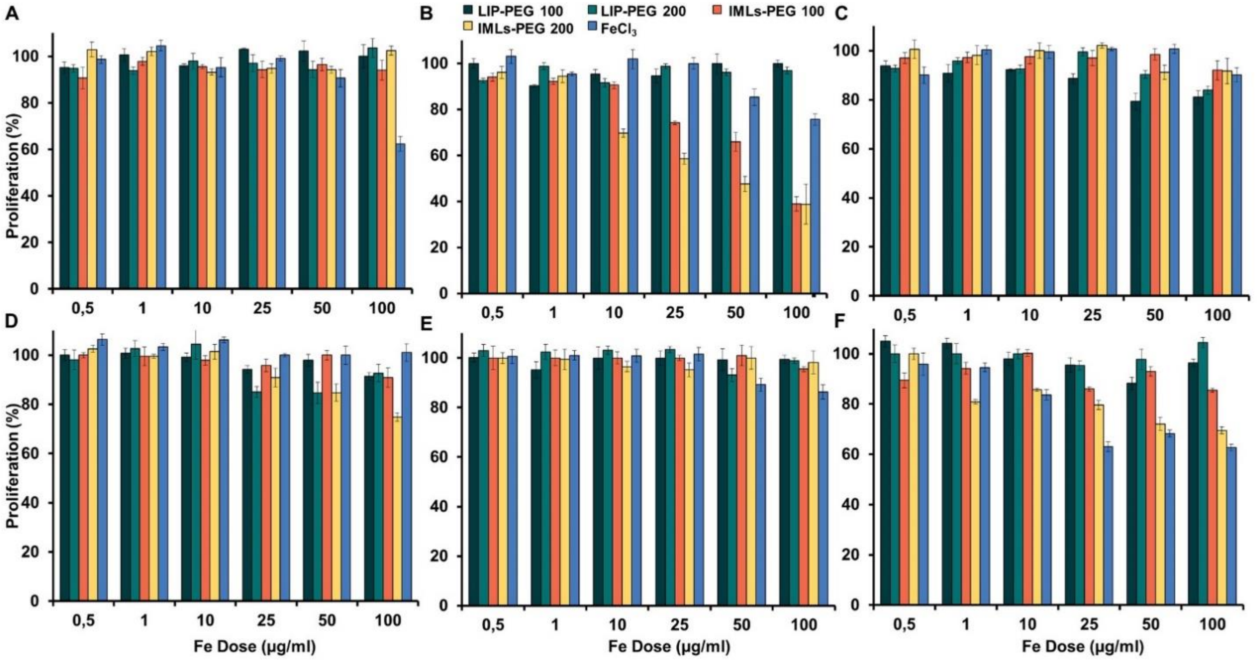

Figure S3. Cell proliferation assay of colon cell lines treated with $FeCl_3$, LIP-PEG and IMLs-PEG. The cell lines T84 (A), CCD18 (B), SW480 (C), HCT15 (D), HT29 (E) and MC38 (F) were exposed to increasing concentrations of Fe from 0.5 to 100 µg/mL from the different NPs for 72 h. The graphs represent the percentages of proliferation of all the cell lines obtained using the SRB assay. Data represent the mean values ± SD of triplicate cultures.

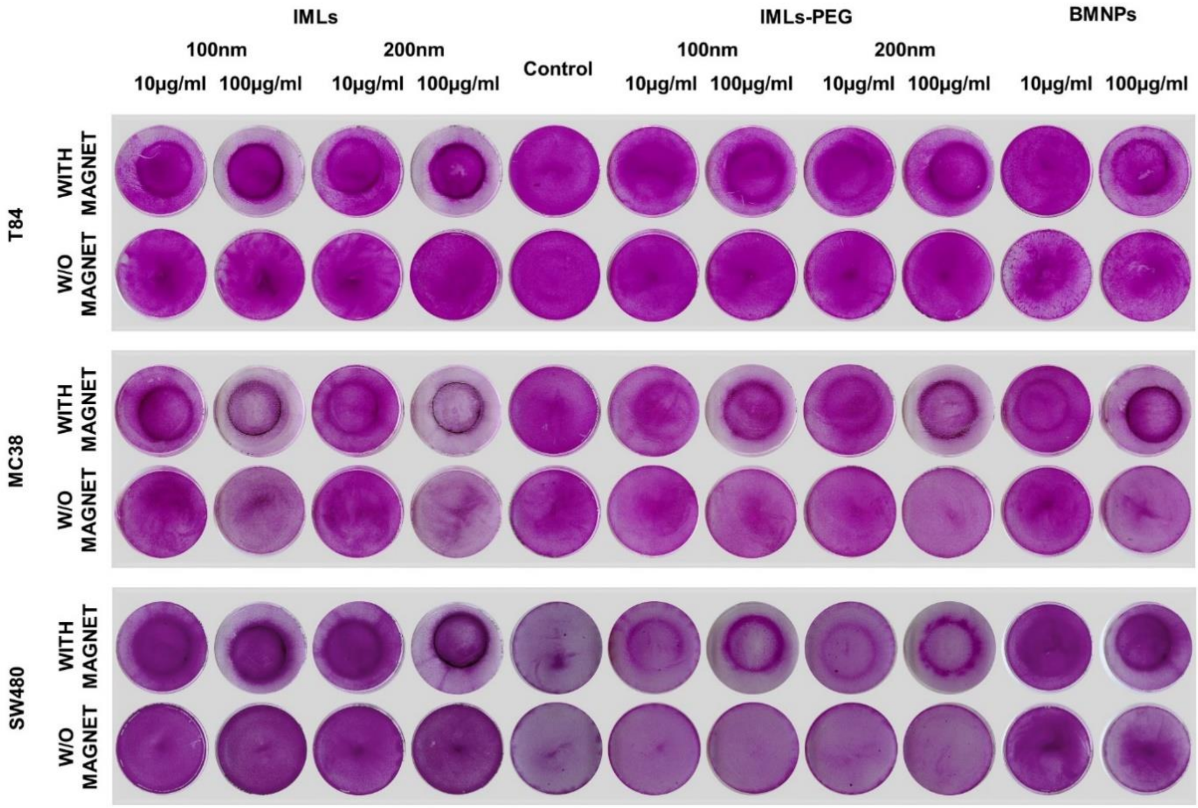

Figure S4. Cell migration assay. Representative image of colon cancer cells (T84, MC38 and SW480) exposed to different concentrations of IMLs, BMNPs and $FeCl_3$ and stained with SRB. Migration of the cells after treatments was evaluated in the presence or absence of a magnet.

Table S1. Average size and potential zeta values of different magnetoliposomes formulations.

Sample	Size (nm)	PDI	Zeta Potential (mV)
BMLs 200	227 ± 4	0.38 ± 0.01	-20.4 ± 0.9
BMLs 100	98 ± 2	0.253 ± 0.009	
Oxa-BMLs-PEG 200	235 ± 5	0.36 ± 0.02	-27.3 ± 0.4
Oxa-BMLs-PEG 100	134.1 ± 0.9	0.162 ± 0.004	
